# Novel role of cannabinoid receptor 2 in inhibiting EGF/EGFR and IGF-I/IGF-IR pathways in breast cancer

**DOI:** 10.18632/oncotarget.9408

**Published:** 2016-05-17

**Authors:** Mohamad Elbaz, Dinesh Ahirwar, Janani Ravi, Mohd W. Nasser, Ramesh K. Ganju

**Affiliations:** ^1^ Department of Pathology and The Comprehensive Cancer Center, The Ohio State University, Wexner Medical Center, Columbus, OH, USA; ^2^ Department of Pharmacology, Pharmacy School, Helwan University, Helwan, Egypt

**Keywords:** EGFR, IGF-IR, CNR2

## Abstract

Breast cancer is the second leading cause of cancer deaths among women. Cannabinoid receptor 2 (CNR2 or CB2) is an integral part of the endocannabinoid system. Although CNR2 is highly expressed in the breast cancer tissues as well as breast cancer cell lines, its functional role in breast tumorigenesis is not well understood. We observed that estrogen receptor-α negative (ERα-) breast cancer cells highly express epidermal growth factor receptor (EGFR) as well as insulin-like growth factor-I receptor (IGF-IR). We also observed IGF-IR upregulation in ERα+ breast cancer cells. In addition, we found that higher CNR2 expression correlates with better recurrence free survival in ERα- and ERα+ breast cancer patients. Therefore, we analyzed the role of CNR2 specific agonist (JWH-015) on EGF and/or IGF-I-induced tumorigenic events in ERα- and ERα+ breast cancers. Our studies showed that CNR2 activation inhibited EGF and IGF-I-induced migration and invasion of ERα+ and ERα- breast cancer cells. At the molecular level, JWH-015 inhibited EGFR and IGF-IR activation and their downstream targets STAT3, AKT, ERK, NF-kB and matrix metalloproteinases (MMPs). *In vivo* studies showed that JWH-015 significantly reduced breast cancer growth in ERα+ and ERα- breast cancer mouse models. Furthermore, we found that the tumors derived from JWH-015-treated mice showed reduced activation of EGFR and IGF-IR and their downstream targets. In conclusion, we show that CNR2 activation suppresses breast cancer through novel mechanisms by inhibiting EGF/EGFR and IGF-I/IGF-IR signaling axes.

## INTRODUCTION

Cannabinoids are classified into 3 types: phytocannabinoids, which are extracted from *cannabis sativa*, endocannabinoids that are produced in the animal body, and synthetic cannabinoids that are synthesized in the laboratories. Endocannabinoid system consists of cannabinoids, cannabinoid receptors and the enzymes that metabolize the endogenous cannabinoids, such as fatty acid amide hydrolase (FAAH) and monoacyl glycerol lipase (MAGL). Cannabinoids act primarily on G-protein coupled receptors (GPCRs): cannabinoid receptors 1 and 2 (CNR1 and CNR2) [1]. CNR2 (CB2) is a component of the endocanabinoid system. Different endocannabinoids such as anandamide (AEA) have been shown to possess affinity towards it [2]. Endocannabinoid compounds have been shown to inhibit breast cancer cell proliferation and migration through different mechnisms [3]. Synthetic and phytocannabinoids have also been shown to possess anti-proliferative and anti-invasive potentials *in vitro* and *in vivo* [4–7]. In the present study, we analyzed the anti-tumorigenic properties of CNR2 specific agonist synthetic cannabinoid (JWH-015) against different breast cancer subtypes.

CNR2 is highly expressed in most immune cells [8, 9]. Different studies suggest a potential role of CNR2 in immune system. CNR2 knock out mice showed significantly less memory B-cells, CD4^+^ and CD8^+^ T-cells compared to heterozygous mice [10]. In addition to immune cells, CNR2 has also been shown to be expressed in breast cancer tissues and cell lines [6]. CNR2 specific agonists (JWH-015 and JWH-133) inhibit growth through different mechanism including inhibition of CXCL12/CXCR4 axis, cyclo-oxygenase-2 (COX-2) expression and induction of apoptosis in different cancer types [1, 5, 6]. Although there is a strong evidence of the anti-tumorogenic properties of CNR2 agonists, not much is known about the role of CNR2 activation on different growth factor receptor, such as epidermal growth factor receptor (EGFR) and insulin-like growth factor 1 receptor (IGF-IR)-mediated tumorigenic events in breast cancer.

Overexpression of EGFR and IGF-IR, lack of hormonal targeted cancer therapy, low survival rate and poor patient prognosis are hallmark features of the estrogen receptor alpha-negative (ERα-) breast cancer subtype [11–15]. The standard therapy for ERα- breast cancer using anthracyclins, taxanes and platinum compounds has shown unresponsiveness and rapid development of resistance [16–20]. Insulin growth factor receptor (IGF-IR) is predominantly activated in ERα+ as well as ERα- breast cancer subtypes [21, 22]. IGF-I has shown a great importance in breast cancer progression through its anti-apoptotic, mitogenic, and invasive potential in both ERα+ and ERα- breast cancer cells [23–25]. ERα+ breast cancer cells are associated with high expression of IGF-IR [26]. IGF-IR activation is, in turn, associated with increased invasion, secretion of metalloproteinases (MMPs) and activation of EMT process in ERα+ cells [24]. ERα- breast cancer cells are also associated with hyperactivation of IGF-IR [22]. IGF-IR activation and phosphorylation is associated with poor prognosis in many breast cancer subtypes including ERα+ and ERα- subtypes [27].

In this study, we analyzed the effect of the CNR2 activation on different tumorigenic related events. First, we analyzed the effect of CNR2 specific agonist on EGF-induced tumorigenic events in ERα- breast cancer cells. Second we analyzed the effect of CNR2 activation on IGF-I induced tumorigenesis in ERα+ and ERα- breast cancer cells. We also studied the effect of CNR2 activation on breast cancer growth using ERα+ and ERα- breast cancer mouse model systems.

## RESULTS

### CNR2 is expressed in ERα- and ERα+ breast cancer subtypes and associated with better prognosis

**Figure 1:**
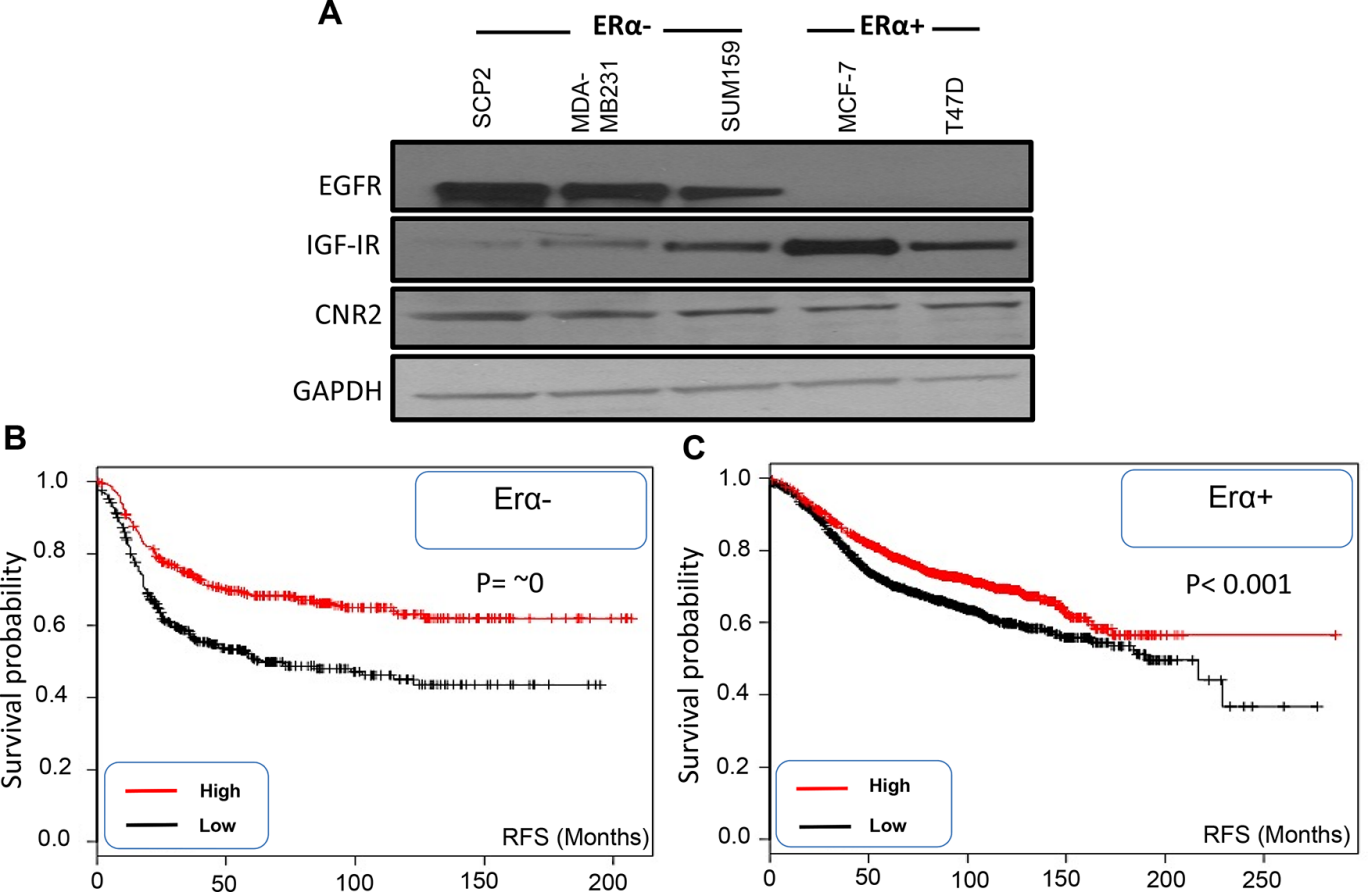
Expression of EGFR, IGF-IR and CNR2 proteins in ERα- and ERα+ breast cancer cells and correlation of CNR2 to breast cancer patient prognosis **A.** Western blot image shows expression of EGFR, IGF-IR and CNR2 proteins in different ERα- (SCP2, MDA-MB231 and SUM159) and ERα+ (MCF-7 and T47D) breast cancer cell lines. GAPDH was used as a loading control. **B.** Kaplan Meier blot showing recurrence free survival (RFS) of high/low expressing CNR2 breast cancer patients of ERα- subtype. P value = ~ zero. **C.** Kaplan Meier blot showing recurrence free survival (RFS) of high/low expressing CNR2 breast cancer patients of ERα+ subtype. P value = 1e^−05^.

In this study, we analyzed CNR2, IGF-IR and EGFR expression in ERα- and ERα+ breast cancer cells. As shown, EGFR is highly expressed in different ERα- cell lines compared to ERα+ breast cancer cell lines however; CNR2 is highly expressed in both breast cancer subtypes (Figure 1-A). IGF-IR is highly expressed in different ERα+ cell lines especially MCF-7 cells; it is also expressed in ERα- breast cancer cells but it might be at lower levels (Figure 1-A). Thereafter, we used SUM159 and MDA-MB231 cells as representatives of ERα- breast cancer cells [28] and MCF-7 as a representative of ERα+ breast cancer cells [29]. In order to analyze the correlation of CNR2 to the prognosis of the breast cancer patients, we used Kaplan Meier publically available datasets. We observed that higher CNR2 expression correlates with significantly better recurrence free survival (RFS) in ERα-, basal, ERα+ and luminal A breast cancer patients (Figure 1B-1C and Supp. Figure 1A-1B).

These results show that EGFR is highly expressed in ERα- breast cancer cells whereas; IGF-IR is highly expressed in ERα+ breast cancer cells. CNR2 is expressed in both ERα+ and ERα- breast cancer cells and associated with better prognosis in both subtypes.

### CNR2 activation inhibits EGF-induced tumorigenic events in ERα- breast cancer cells

**Figure 2:**
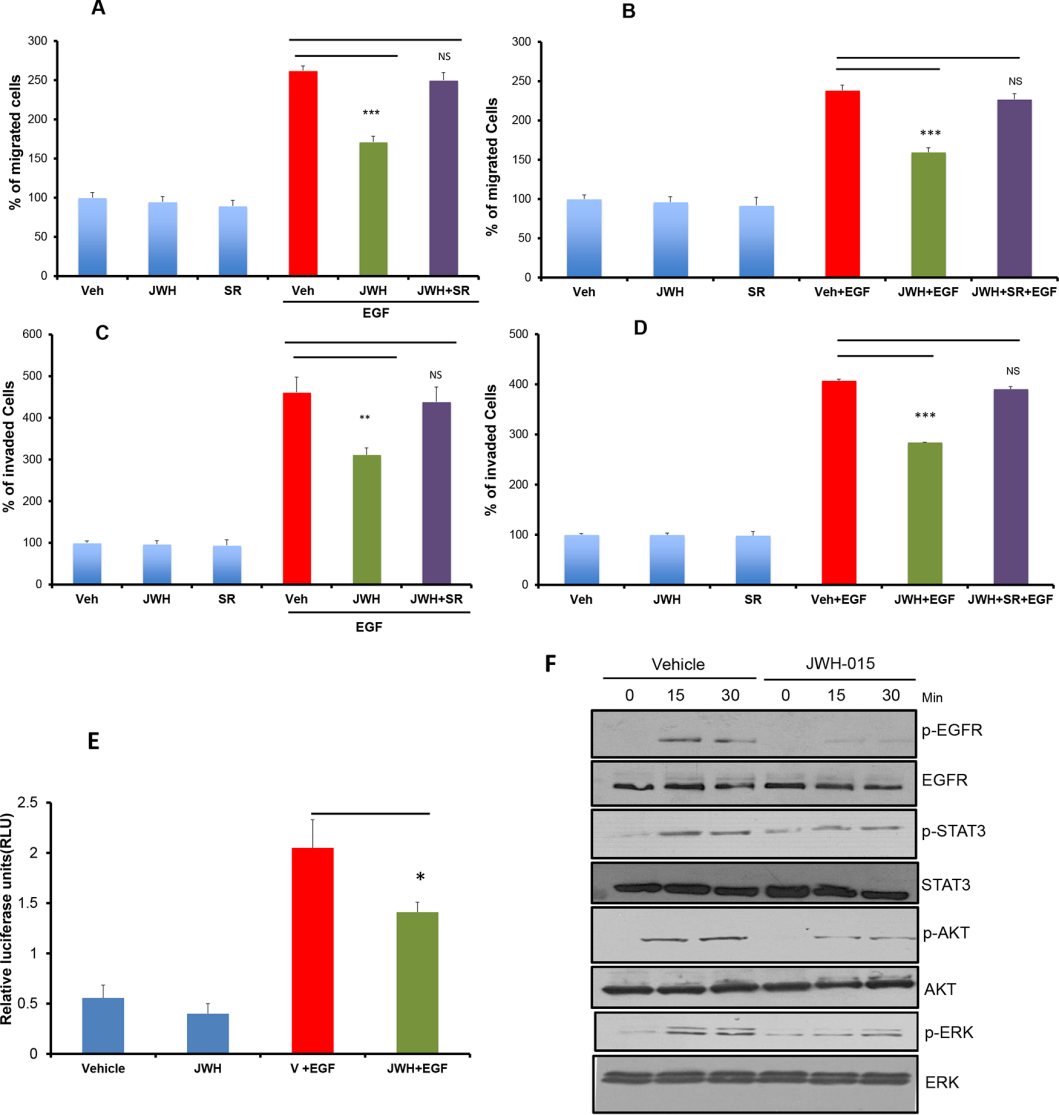
CNR2 activation inhibits EGF/EGFR signaling in ERα- breast cancer cells SUM159 **A.** or MDA-MB231 **B.** cells were treated with JWH-015 for 24 hours and subjected to transwell migration assay in presence or absence of EGF (100 ng/ml) and specific CNR2 blocker SR-144528. SUM159 **C.** or MDA-MB231 **D.** cells were treated with JWH-015 for 24 hours and subjected to transwell invasion assay in presence or absence of EGF (100 ng/ml) and specific CNR2 blocker SR-144528. Number of migrated or invaded cells were counted and plotted as % of control. NS = non significant. **E.** SUM159 cells were treated with vehicle or JWH-015 in presence or absence of EGF and subjected to NF-kB luciferase reporter assay. **F.** SUM159 cells were treated with vehicle or JWH-015, stimulated with EGF (100 ng/ml) for 15 or 30 minutes then cell lysates were used for western blot analysis for the indicated proteins.

To analyze the possible role of CNR2 activation on EGF/EGFR pathway, we studied the effect CNR2 specific agonist (JWH-015) [1] on EGF-induced tumorigenic events in ERα- cell lines. We found that JWH-015 significantly inhibits EGF-induced migration as well as invasion of SUM159 and MDA-MB231 cells (Figure 2A:2D). NF-kB activation is crucial for cancer cells to promote their migration and invasive potentials especially for ERα- cells [30, 31]. Moreover, EGFR overexpression strongly correlates with NF-kB activation [32]. Therefore, we analyzed the effect of CNR2 agonist on NF-kB activation by using luciferase reporter assay. As shown, JWH-015 significantly inhibits NF-kB active units after EGF stimulation (Figure 2E). To test the specificity of JWH-015 towards CNR2, we used CNR2-specific antagonist (SR144528), and we found that CNR2 blocking significantly abrogated JWH-015-mediated inhibition of migration and invasion of SUM159 and MDA-MB231 cells after EGF stimulation (Figure 2A:2D). To further analyze the molecular mechanism, we investigated CNR2 agonist's effect on EGF/EGFR axis. As shown, JWH-015 inhibits EGFR activation and its downstream targets STAT3, AKT and ERK (Figure 2F).

These results indicate that CNR2 triggering has the ability to inhibit EGFR activation and its downstream targets and therefore suppresses EGF-induced tumorigenic events in ERα- breast cancer cells.

### CNR2 activation inhibits IGF-I-induced tumorigenic events in ERα- breast cancer cells

**Figure 3:**
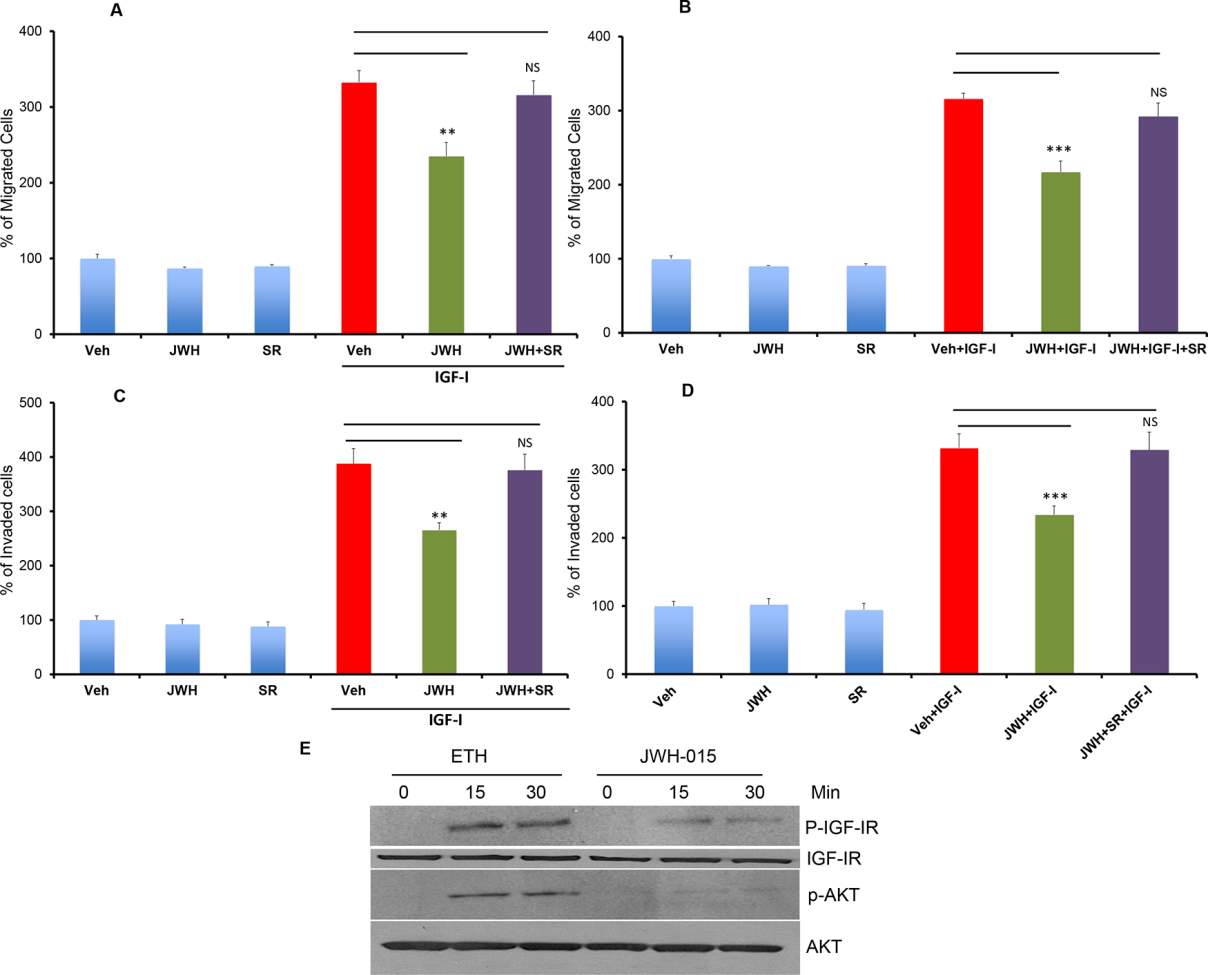
CNR2 activation inhibits IGF/IGF-IR signaling in ERα- breast cancer cells SUM159 **A.** or MDA-MB231 **B.** cells were treated with JWH-015 for 24 hours and subjected to transwell migration assay in presence or absence of IGF-I (50 ng/ml) and specific CNR2 blocker SR-144528. SUM159 **C.** or MDA-MB231 **D.** cells were treated with JWH-015 for 24 hours and subjected to transwell invasion assay in presence or absence of IGF-I (50 ng/ml) and specific CNR2 blocker SR-144528. Number of migrated or invaded cells were counted and plotted as % of control. NS = non significant. **E.** SUM159 cells were treated with vehicle or JWH-015, stimulated with IGF-I (50 ng/ml) for 15 or 30 minutes then cell lysates were used for western blot analysis for the indicated proteins.

ERα- breast cancer cells are also associated with hyperactivation of IGF-IR [22]. IGF-IR activation and phosphorylation is associated with poor prognosis in many breast cancer subtypes including ERα+ and ERα- subtypes [27]. We have shown that ERα- breast cancer cells also express IGF-IR (Figure 1A). Therefore, we investigated the role of JWH-015 on IGF-I-induced tumorigenic events in SUM159 and MDA-MB231 cells (ERα-). We found that JWH-015 significantly inhibits IGF-I-induced migration as well as invasion of SUM159 and MDA-MB231 cells (Figure 3A:3D). At the molecular level, we found that JWH-015 inhibits IGF-I-induced activation of IGF-IR and its downstream target AKT in SUM159 cells (Figure 3E and Supp. Figure 2A-2B). We also found that JWH-015 inhibits secretion of metalloproteinase-2 (MMP2) in SUM-159 cells (Supp. Figure 4). To confirm the specificity of JWH-015 towards CNR2 in these studies, we used a CNR2-specific antagonist (SR144528), and we found that CNR2 blocking has significantly abrogated JWH-015 potential in inhibition of migration and invasion induced by IGF-I in SUM159 and MDA-MB231 cells (Figure 3A:3D). These results indicate that CNR2 activation has the ability to inhibit IGF-IR activation and its downstream key molecule (AKT), which might explain the inhibitory effect of JWH-015 on IGF-I-induced migration and invasion in ERα- cells.

**Figure 4:**
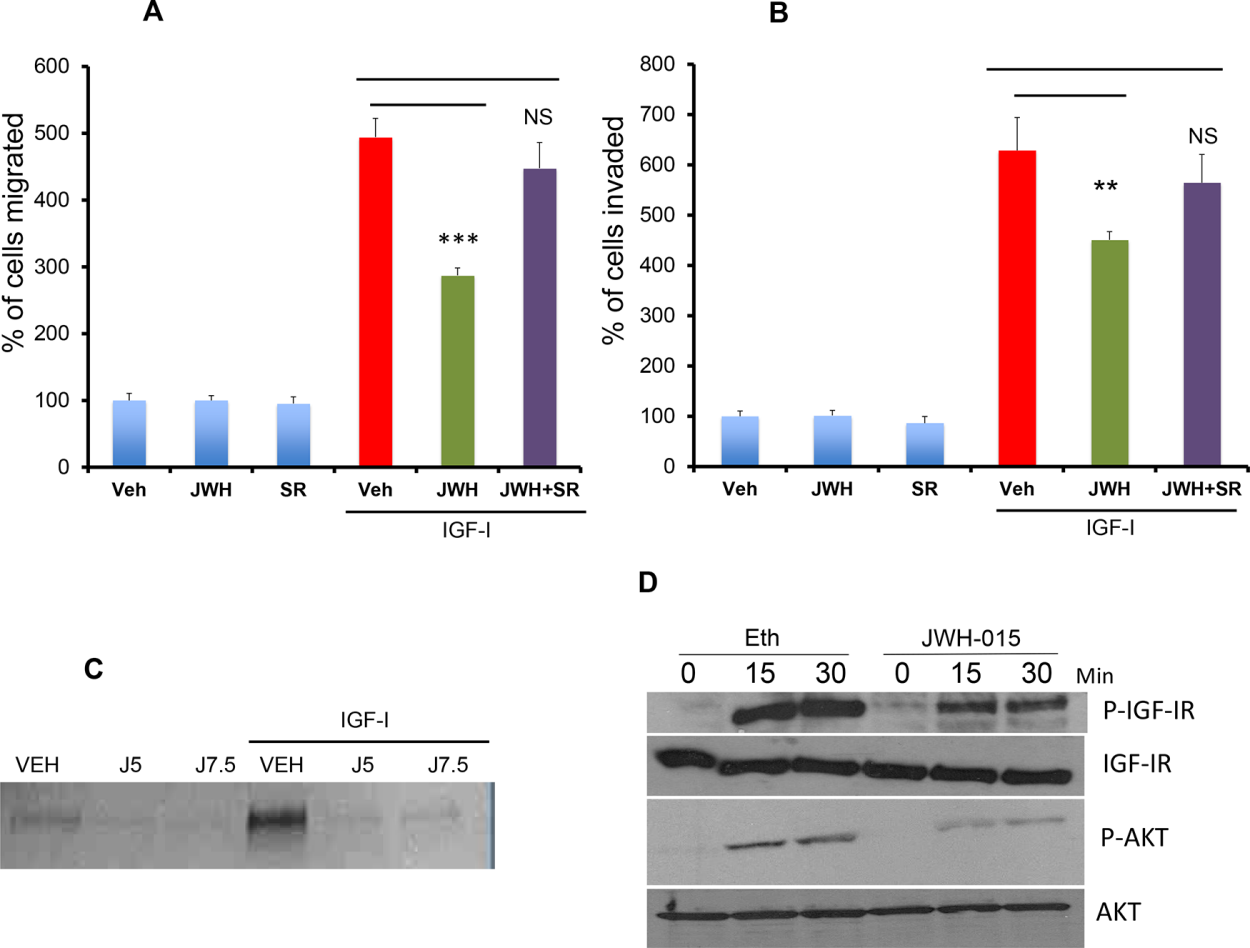
JWH-015 inhibits IGF-I/IGF-IR signaling in ERα+ breast cancer cells **A.** MCF-7 cells were treated with JWH-015 for 24 hours and subjected to transwell migration assay in presence or absence of IGF-I (50 ng/ml) and specific CNR2 blocker SR-144528. **B.** MCF-7 cells were treated with JWH-015 for 24 hours and subjected to transwell invasion assay in presence or absence of IGF-I (50 ng/ml) and specific CNR2 blocker SR-144528. Number of migrated or invaded cells were counted and plotted as % of control. NS = non significant. **C.** MCF-7 cells were treated with vehicle or JWH-015 5 or 7.5 μM in presence or absence of IGF-I and the collected conditioned media was subjected to gelatin zymography to detect MMP-9. **D.** MCF-7 cells were treated with vehicle or JWH-015, stimulated with IGF-I (50 ng/ml) for 15 or 30 minutes then cell lysates were used for western blot analysis for the indicated proteins.

### CNR2 activation inhibits IGF-I-induced tumorigenic events in ERα+ breast cancer cells

ERα+ breast cancer cells are associated with hyperactivation of IGF-IR [26]. IGF-IR activation is, in turn, associated with increased invasion, secretion of metalloproteinases (MMPs) and activation of EMT process in ERα+ cells [24].

Therefore, in this study, we analyzed the role of CNR2 activation on IGF-I-induced tumorigenic events in ERα+ breast cancer cells. In MCF-7 cells (ERα+), we found that JWH-015 significantly inhibits IGF-I-induced migration and invasion (Figure 4A-4B). We subsequently analyzed the molecular mechanism through investigation CNR2 agonist's effect on IGF-I/IGF-IR pathway. As shown, JWH-015 inhibits the activation of IGF-IR and its downstream target AKT (Figure 4D and Supp. Figure 2C-2D). To investigate the role of CNR2 activation on IGF-I-induced secretion of metalloproteinases, we used zymogram technique. Interestingly, we found that JWH-015 inhibits IGF-I-induced secretion of metalloproteinase-9 (MMP9) in MCF-7 cells (Figure 4C). To confirm the specificity of JWH-015 towards CNR2 in these studies, we used a CNR2-specific antagonist (SR144528), and we found that CNR2 blocking has significantly abrogated JWH-015-potential in inhibition of migration and invasion induced by IGF-I in MCF-7 and SUM159 cells (Figure 4A-4B).

These results indicate that CNR2 activation has the ability to inhibit IGF-IR activation and its downstream target. It also inhibits IGF-I-induced secretion of MMP9, which might explain the inhibitory effect of JWH-015 on IGF-I- induced migration and invasion in ERα+ cells.

### JWH-015 inhibits EGF/EGFR and IGF-I/IGF-IR axes in ERα- and ERα+ orthotopic mouse models

**Figure 5:**
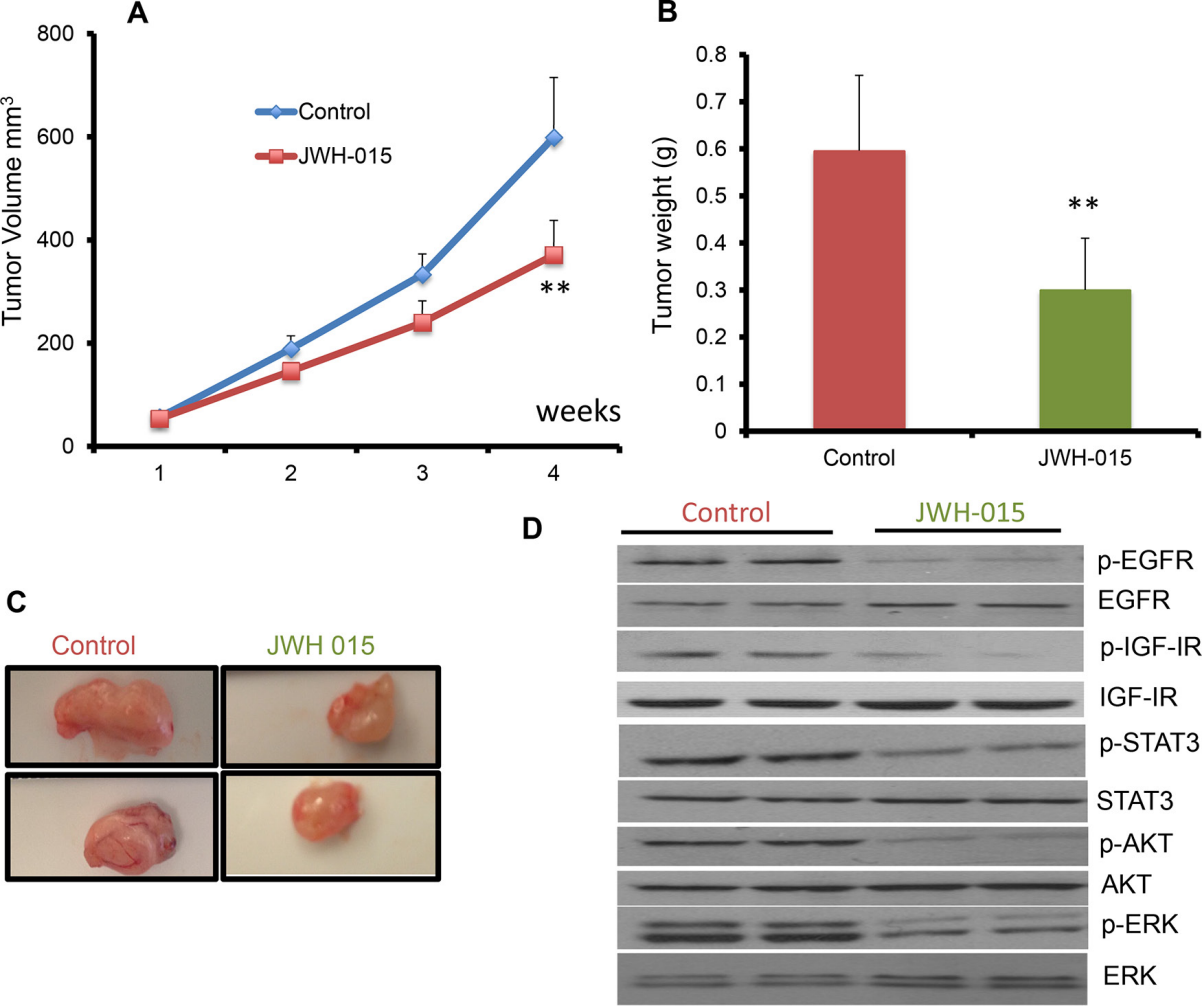
JWH-015 suppresses ERα- breast cancer growth *in vivo* by inhibiting EGF/EGFR and IGF-I/IGF-IR signaling pathways **A.** Tumor volume measurements of orthotopically injected nude mice with SUM159 cells were assessed every week for control and treated groups. **B.** Tumor weight of vehicle-treated or JWH-015-treated nude mice was determined at the euthanasia day. **C.** Representative photographs showing tumors dissected from control and treated groups. **D.** Western blot images of the tumor lysates of the control and treated groups showing the protein expression of phospho-EGFR, phospho-IGF-IR, phospho-STAT3 phospho-ERK and phosphor-AKT (p-EGFR, p-IGF-IR, p-STAT3, p-ERK and p-AKT) and total EGFR, IGF-IR, STAT3, ERK and AKT (EGFR, IGF-IR, STAT3, ERK and AKT).

**Figure 6:**
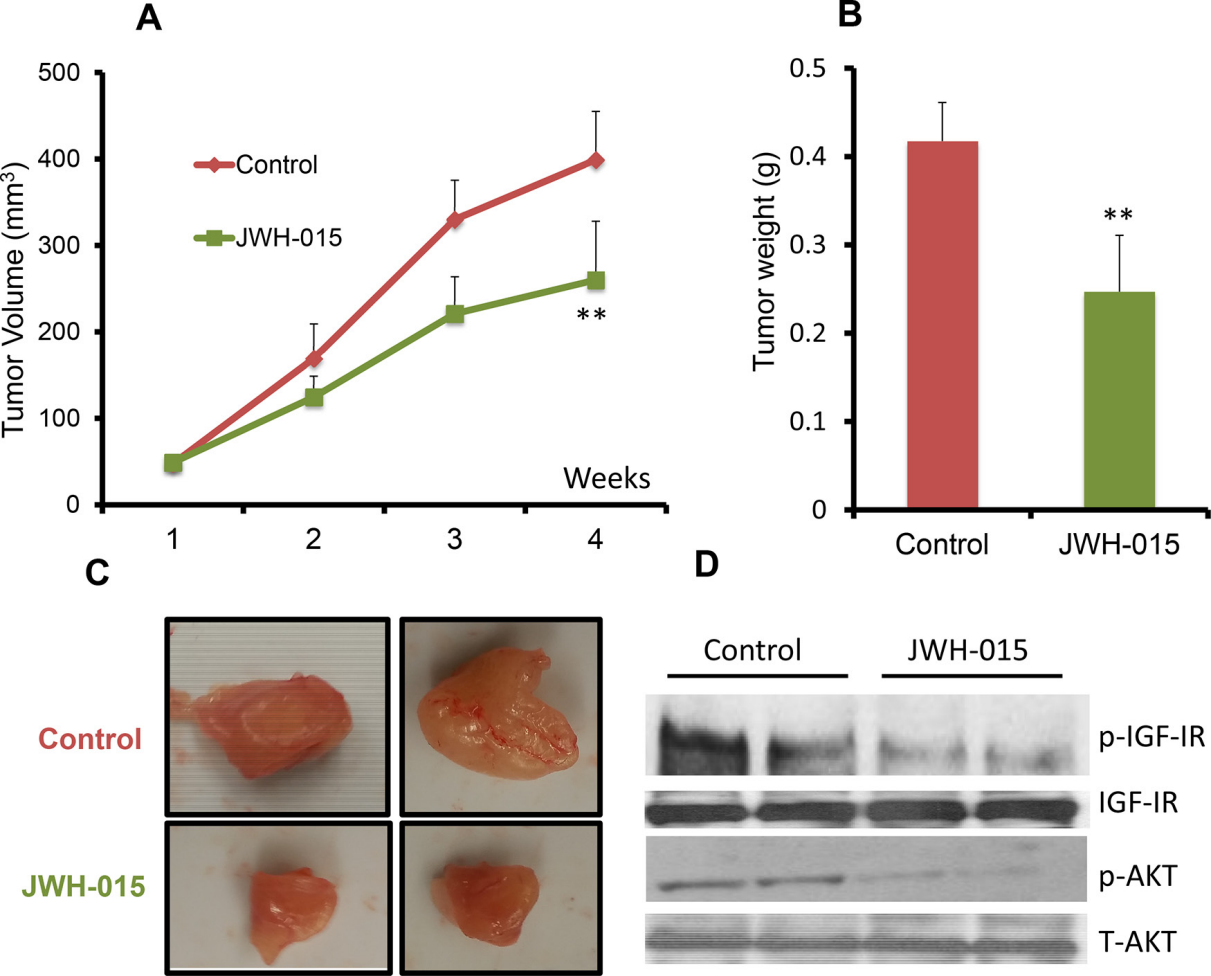
JWH-015 suppresses ERα+ breast cancer growth *in vivo* **A.** Tumor volume measurements of orthotopically injected nude mice with MCF-7 cells were assessed every week for control and treated groups. **B.** Tumor weight of vehicle-treated or JWH-015-treated nude mice was determined at the euthanasia day. **C.** Representative photographs showing tumors dissected from control and treated groups. **D.** Western blot images of the tumor lysates of the control and treated groups showing the protein expression of phospho-IGF-IR and phosphor-AKT (p-IGF-IR and p-AKT) and total IGF-IR and AKT (IGF-IR and AKT).

In order to analyze the anti-tumorigenic potential of CNR2 specific agonist on ERα- cells *in vivo*, we injected nude mice orthotopically with SUM159 cells. After the tumor became palpable, the mice were treated, through peri-tumoral injection, with either vehicle or JWH-015 (10 mg/Kg) for 4 weeks. The tumors were then harvested for analysis. As shown, JWH-015-treated group has less tumor volume and tumor weight compared to vehicle-treated group (Figure 5A:5C). We analyzed the tumor lysates to find out the ability of CNR2 activation to inhibit EGFR and IGF-IR activation *in vivo* and found that the JWH-015-treated group has less activation of EGFR, IGF-IR, STAT3, AKT and ERK proteins compared to control group (Figure 5D and Supp. Figure 3A:3E). In order to analyze the anti-tumorigenic potential of CNR2 specific agonist on ERα+ cells *in vivo*, we injected nude mice orthotopically with MCF-7 cells. After the tumor became palpable, the mice were treated, through peri-tumoral injection, with either vehicle or JWH-015 (10 mg/Kg) for 4 weeks. The tumors were then harvested for analysis. As shown, JWH-015-treated group has less tumor volume and tumor weight compared to vehicle-treated group (Figure 6A:6C). We also analyzed the tumor lysates and found that the JWH-015-treated group has less activation of IGF-IR and AKT proteins compared to the control group (Figure 6D and Supp. Figure 3F-3G). These results suggest that CNR2 activation has the ability to inhibit breast cancer growth through inhibition of EGF/EGFR as well as IGF-I/IGF-IR pathways and their downstream targets.

## DISCUSSION

CNR2 has been shown to be expressed in different cancer cells, including breast cancer [6, 33, 34]. Although CNR2 activation has shown significant anti-tumorigenic properties, its functional role in inhibiting breast cancer is not well understood. In this study we showed that CNR2 is expressed in both ERα- and ERα+ breast cancer cells. ERα- breast cancer is characterized by its high proliferative and metastatic potential and poor prognosis. ERα+ breast cancer represents most of all breast cancers cases and is also associated with poor prognosis, low overall survival particularly when associated with lymph node metastasis [35–37]. In the present study, we elucidated the anti-tumor role of CNR2 in ERα+ and ERα- breast cancer cells. Following CNR2 activation, we observed inhibition of breast cancer growth, suppression of EGF/EGFR as well as IGF-I/IGF-IR signaling pathways and their related tumorigenic events *in vitro* and *in vivo*.

Since EGFR is upregulated in ERα- breast cancer subtype, EGFR inhibitors have been used as a possible therapeutic option. However, EGFR inhibitors usually gain rapid resistance and they eventually fail [38]. Therefore, it is crucial to identify other small molecules that can achieve the interruption of EGF/EGFR axis by different, possibly indirect, ways. In our present study, we showed that CNR2 activation by CNR2 specific agonist (JWH-015) inhibits EGF/EGFR signaling transduction pathway's activation in ERα- cells *in vitro* and *in vivo*. This pathway involves important key molecules STAT3, AKT and ERK and NF-kB, which are important in cancer cell survival, proliferation, migration and invasion. These results explain the ability of JWH-015 to inhibit EGF-induced migration and invasion as well as inhibition of phosphorylation of EGFR, AKT and ERK proteins in ERα- cells *in vitro* and *in vivo*. In our study, we found that JWH-015 also inhibited EGF-induced NF-kB activation in ERα- breast cancer cells. NF-kB is highly active in ERα- breast cancer cells and it enhances breast cancer cell's migration, invasion and metastasis through different mechanisms [39]. It promotes the expression of several pro-tumorigenic genes such as CXCR4, which is crucial for cancer cell migration and invasion [30, 40]. Furthermore, NF-kB activation is important for epithelial to mesenchymal transition (EMT) process [41]. Our studies are in accordance with previous studies, which showed that CNR2 agonists inhibit the tumor growth and metastasis through induction of apoptosis and inhibition of EGFR activation in non-small cell lung cancer (NSCLC) [5]. Our findings might explain the observation, which shows that higher CNR2 expression is significantly correlated with better prognosis in ERα- patients.

A common problem for ERα+ breast cancer is the resistance to the anti-estrogen therapy [42, 43]. After gaining resistance to the estrogen deprivation, ERα+ cells show upregulation and activation of IGF-I/IGF-IR and PI3K/AKT signaling [44]. Furthermore, IGF-IR inhibitors also gain rapid resistance [45, 46]. It is urgently needed to find other options for targeting IGF-I/IGF-IR in ERα+ and ERα- cells rather than conventional direct therapies. Therefore, we studied the effect of CNR2 agonist on IGF-I-induced tumorigenic events. Our results showed that JWH-015 inhibits phosphorylation and activation of IGF-IR and its downstream target AKT *in vitro* and *in vivo*. This might explain its ability to inhibit IGF-I-induced migration in ERα+ and ERα- cells. The inhibition of IGF-I-induced secretion of MMP-9 by JWH-015 might also explain the ability of CNR2 agonist to inhibit IGF-I-induced invasion in ERα+ cells. These findings might explain our observation that show that higher CNR2 expression is significantly correlated with better prognosis in ERα- as well as ERα+ patients.

In this study, we showed that CNR2 activation inhibits the activation of EGFR and IGF-IR receptor tyrosine kinases (RTKs), however the mechanism by which CNR2 inhibits these RTKs activation is not known. Importantly, Pérez-Gómez et al; have recently reported that CNR2 cross talks with HER-2 through forming a heterodimer. In addition, CNR2 has been shown to cross talks with other G-protein coupled receptors (GPCR) such as CXCR4 through forming heterodimers and thereby inhibiting its activity [5, 47]. Therefore, there is possibility of similar direct interaction between CNR2 with EGFR and IGF-IR. There is also a probability that the cross-talk between CNR2 and these RTKs occurs through indirect way. Previous studies showed that MMP inhibitor inhibits EGFR ligand shedding and secretion that resulted in less activation of EGFR [48]. Since we have shown that JWH-015 inhibits MMP secretion, this may lead to less shedding and secretion of EGFR ligands and eventually less activation of EGFR and its downstream signaling.

**Figure 7:**
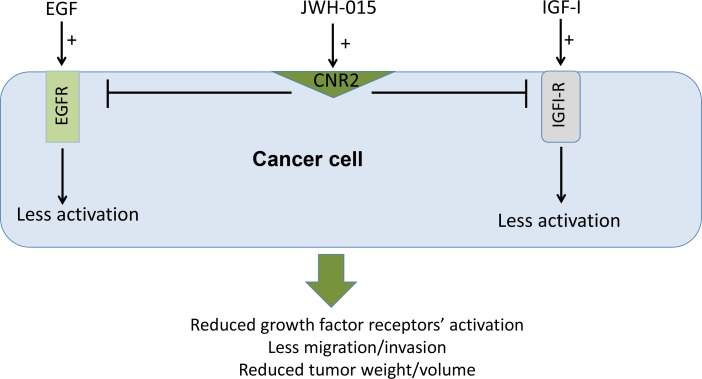
Schematic representation of the anti-tumor role of CNR2 activation in breast cancer Schematic representation of the direct anti-tumor role of CNR2 activation showing the possible cross-talk between CNR2 receptor and EGFR and IGF-IR.

Overall, we show in this study that CNR2 is an important therapeutic target in ERα- and ERα+ breast cancer subtypes and its higher expression is associated with better prognosis. In addition, we show, for the first time, that CNR2 activation inhibits the activation of EGFR/IGF-IR signaling pathways and EGF/IGF-I-induced tumorigenic events in ERα- and ERα+ breast cancer subtypes *in vitro* and *in vivo* (Figure 7). Our data support the use of CNR2 agonists as an adjuvant therapy for ERα- and ERα+ breast cancer patients.

## MATERIALS AND METHODS

### Reagents and antibodies

Cell culture reagents were obtained from Gibco Laboratories (Grand Island, NY). The following reagents and antibodies used in this study were purchased from different sources: JWH-015 (Tocris Bioscience) is a CNR2 agonist that is used in the whole study, human EGF and IGF-I proteins (Peprotech). Antibodies: CNR2 (Abcam), p-AKT (serine 473), p-STAT3 (serine 727) and p-IGF-IR (Y1135/1136) (Cell Signaling); p-ERK/ERK, p-EGFR (Tyr 1173), GAPDH, STAT3 and AKT (Santa Cruz).

### Cell culture

SUM159 cells were obtained from Dr. Sarmila Majumder (The Ohio State University). MDA-MB 231, T47D and MCF7 cells were purchased from ATCC. SCP2 cells were kindly obtained from Dr. Joan Massagué [49]. Cells were cultured in DMEM containing 10% heat-inactivated fetal bovine serum (FBS), 5-units/mL penicillin, and 5-mg/mL streptomycin.

### Western blot analysis

Cells were plated in 100 cm^2^ dishes and lysed in lysis buffer (RIPA). Tumor samples were homogenized and lysed in RIPA buffer for further analysis. 50 mg of proteins were loaded on 4–12% SDS–polyacrylamide gels (Invitrogen) then transferred to nitrocellulose membranes (BioRad) and blocked with 5% milk. Membranes were incubated overnight with primary antibody, washed, and incubated for 1 h at RT with horseradish peroxidase conjugated secondary antibody. The membranes were then washed and stained using a chemiluminescence system (ECL Amersham Biosciences) and exposed to X-ray film (Kodak).

### Chemotactic assays

Chemotactic assays were performed using transwell chambers (Costar 8.0 mm pore size) as described previously [5, 6, 50]. Briefly, serum starved cells were pretreated with JWH-015 or vehicle for 24 h. Top chambers were loaded with cells in serum free medium (SFM) and bottom chambers contained SFM in the presence or absence of chemo-attractants. Cells that migrated or invaded across the membrane were counted by fixing in 37% formaldehyde and 25% glutaraldehyde in PBS and stained with 0.1% crystal violet in PBS for 30 minutes. Migrated/invaded cells per membrane were detected by light microscopy, counted in 5 fields and the percentage of migration determined.

### Gelatin zymography

Gelatin zymography for collected conditioned media was performed as described earlier [51].

### Luciferase reporter assay

We used NF-kB luciferase reporter assay (Promega) to determine NF-kB activity as described previously [50, 51]. Briefly, NF-kB luciferase constructs containing either wild type or NF-kB vector were transfected in the pretreated cells using lipofectamine 2000 (Invitrogen). For internal control, we co-transfected cells with Renilla luciferase vector. 24 h after transfection, EGF (100 ng/ml) was added and then incubated for another 24 h. Cells were lysed and luciferase assay was performed according to manufacturer's protocol.

### Mouse models

Female nude (NU/NU) mice were purchased from (Charles River Laboratories Inc.). Tumors were formed by orthotopically injecting SUM159 (5 × 10^6^) cells or MCF-7 (5 × 10^6^) in the 4th mammary glands of female nude mice (n=6 mice per group). Mice were randomized when tumors became palpable, and then injected peri-tumorally with JWH-015 (10 mg/kg) or vehicle on alternate days for 4 weeks. Mice with MCF7 cell were subcutaneously injected once a week with 2.5 μg β-estradiol 17-valerate in 50 μL of sesame oil. Tumors were measured every week with external calipers and tumor volume was calculated according to the formula *tumor volume = (length × width^2^ × 0.52)* [1, 52]. The mice did not experience any undesirable effects following administration of vehicle or JWH-015 during the whole period of the experiment.

### Statistical analysis

Results were represented as mean ± SD. Student's t test was used. P<0.05 was considered to be statistically significant. For all graphs, * indicates *P*<0.05, ^*^ indicates *P*<0.01 and ^**^* indicates *P*<0.001.

## SUPPLEMENTARY MATERIALS FIGURES



## Abbreviations

EGFR: epidermal growth factor receptor
IGF-IR: insulin-like growth factor receptor-I
ER: estrogen receptor
CNR2: cannabinoid receptor-2
MMP: matrix metallo-proteinase
