# Supplementary material for: Novel role of cannabinoid receptor 2 in inhibiting EGF/EGFR and IGF-I/IGF-IR pathways in breast cancer
Source: Oncotarget. 2016 May 17;8(18):29668–78. doi: 10.18632/oncotarget.9408 (PMC5444694; doi:10.18632/oncotarget.9408)
Supplement: Supplementary file 1 [file oncotarget-08-29668-s001.pdf]

Novel role of cannabinoid receptor 2 in inhibiting EGF/EGFR and IGF-I/IGF-IR pathways in breast cancer

SUPPLEMENTARY FIGURES

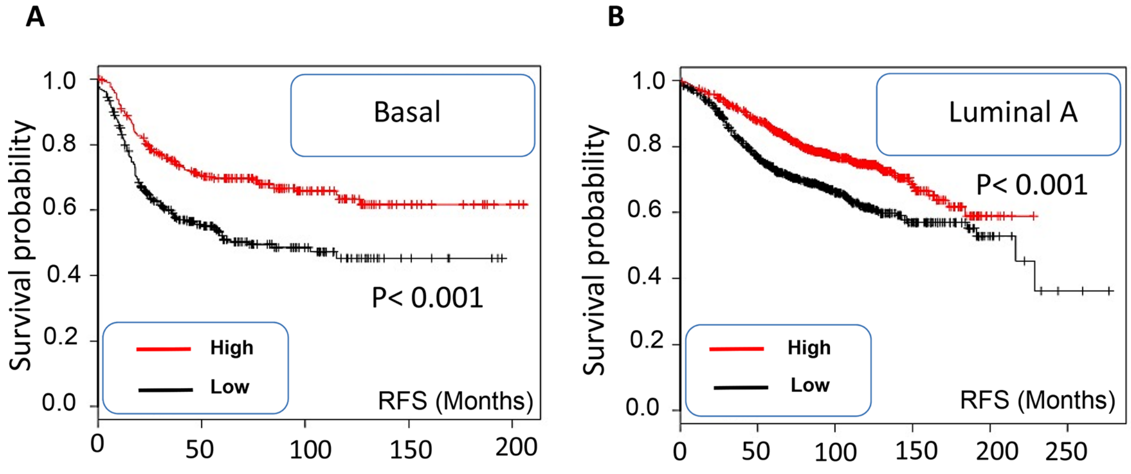

Supplementary Figure S1: Correlation of CNR2 to breast cancer patients' prognosis of Basal and luminal A subtypes. **A.** Kaplan Meier blot showing recurrence free survival (RFS) of high/low expressing CNR2 breast cancer patients of Basal subtype. P value = ~ zero. **B.** Kaplan Meier blot showing recurrence free survival (RFS) of high/low expressing CNR2 breast cancer patients of luminal A subtype. P value = 1e^{-05}.

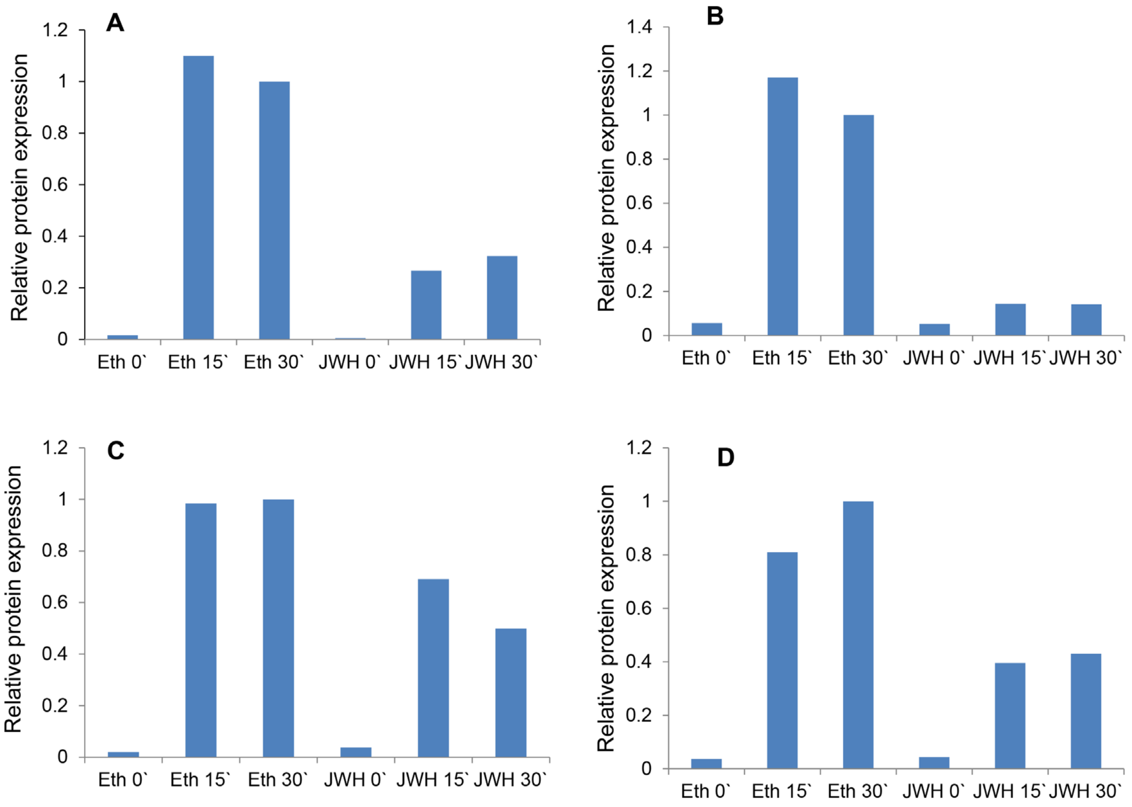

Supplementary Figure S2: Quantification of protein expression showing that JWH-015 inhibits IGF-I induced activation of IGF-IR and AKT. SUM159 cells were treated with JWH-015 and stimulated with IGF-I (50 ng/ml) for 0, 15 or 30 minutes then the cell lysates were used for western blot analysis for the indicated proteins and relative p-IGF-IR/IGF-IR **A.** and relative p-AKT/AKT expressions **B.** have been quantified for every condition. MCF-7 cells were treated with JWH-015 and stimulated with IGF-I (50 ng/ml) for 0, 15 or 30 minutes then the cell lysates were used for western blot analysis for the indicated proteins and relative p-IGF-IR/IGF-IR **C.** and relative p-AKT/AKT **D.** expressions have been quantified for every condition.

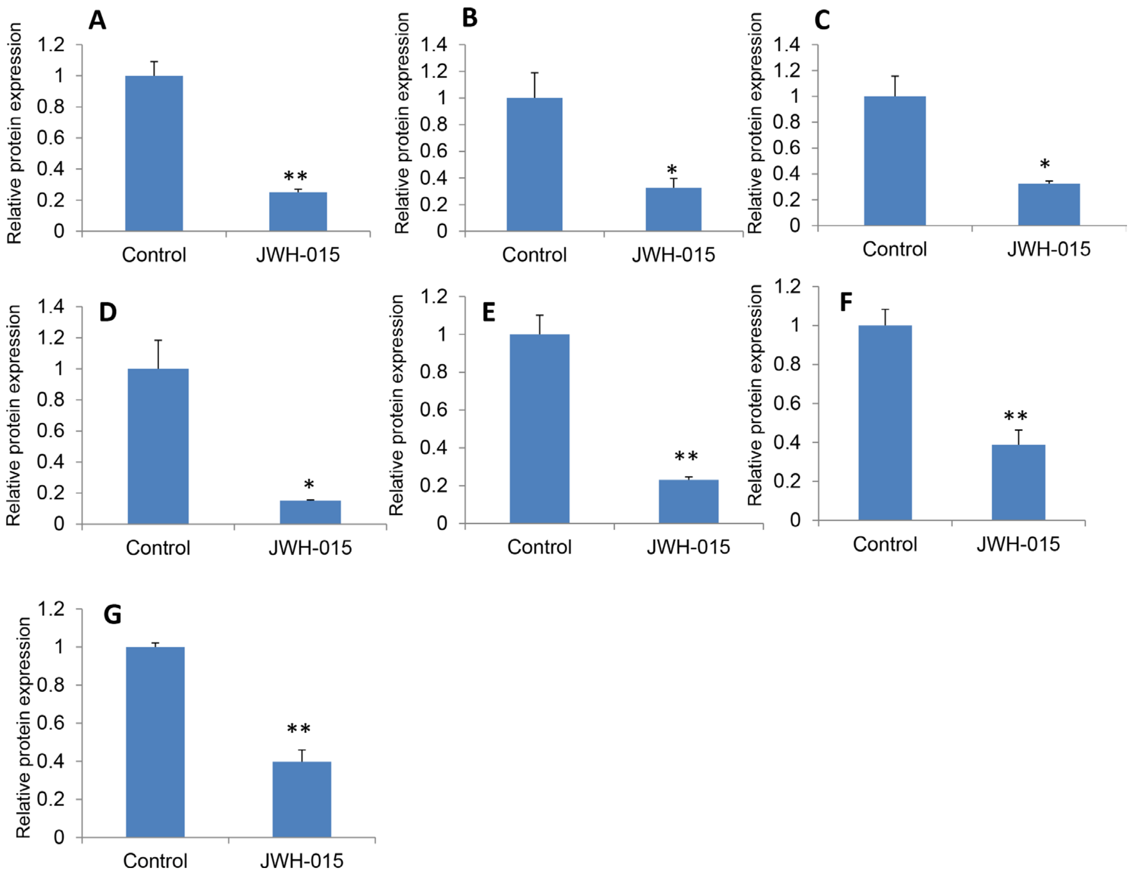

Supplementary Figure S3: Quantification of protein expression in the vehicle and JWH-015-treated tumor lysates. Nude mice were orthotopically injected with SUM159 cells and treated with vehicle or JWH-015 for 4 weeks and the tumors were harvested for western blot protein analysis. Protein expression has been quantified by image-j software for every condition as a relative expression of phosphorylated protein to total protein expression as follows **A.** p-EGFR/EGFR, **B.** p-IGF-IR/IGF-IR, **C.** p-STAT3/ STAT3, **D.** p-AKT/ AKT and **E.** p-ERK / ERK. Nude mice were orthotopically injected with MCF-7 cells and treated with vehicle or JWH-015 for 4 weeks and the tumors were harvested for western blot protein analysis. Protein expression has been quantified by image-j software for every condition as a relative expression of phosphorylated protein to total protein expression as follows **F.** p-IGF-IR/IGF-IR and **G.** p-AKT/ AKT.

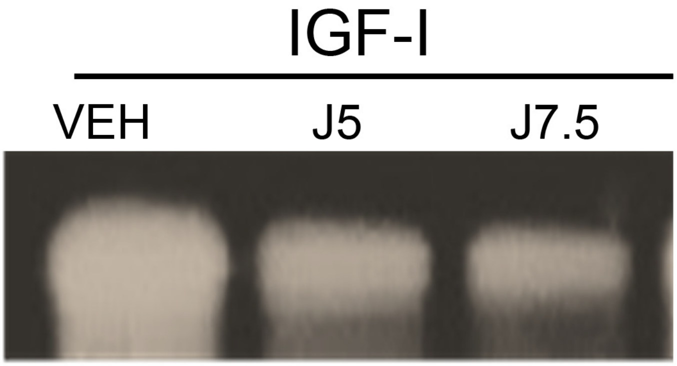

Supplementary Figure S4: JWH-015 inhibits MMP-2 secretion in SUM159 cells. SUM159 cells were treated with vehicle or JWH-015 5 or 7.5 µM in presence of IGF-I and the collected conditioned media were subjected to gelatin zymography to detect MMP-2.

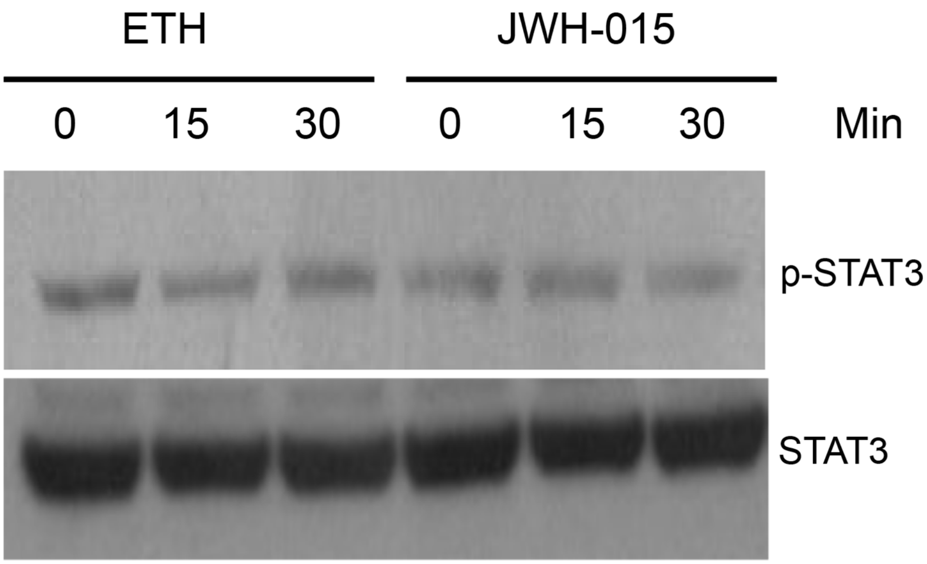

Supplementary Figure S5: JWH-015 does not affect STAT3 activation. SUM159 cells were treated with vehicle or JWH-015 for 24h, then stimulated with IGF-I (50 ng/ml) for 0,15 and 30 minutes and the cells were lysed for detection of the indicated protein by western blot.
